# Assessment of the aesthetic impact and quality of life of home dental bleaching in adult patients

**DOI:** 10.4317/jced.57831

**Published:** 2021-05-01

**Authors:** Rudys-Rodolfo-De Jesus Tavarez, Suellen-Nogueira-Linares Lima, Adriana-Santos Malheiros, Lucas-Lage Menezes, Matheus-Coelho Bandeca, Rita-de Cássia-Mendonça de Miranda, Meire-Coelho Ferreira

**Affiliations:** 1 DDS, PhD. Professor of Ceuma University São Luís MA, Brazil; 2 PhD, Dentistry student, Ceuma University São Luís MA, Brazil

## Abstract

**Background:**

This study aimed to evaluate the impact of home bleaching with 10% carbamide peroxide on the quality of life and aesthetic perception of patients.

**Material and Methods:**

A total of 107 patients between 18 and 38 years of age with good oral and general health and at least one anterior tooth in color A2 or darker were selected. Patients who previously underwent any type of bleaching procedures were excluded. All patients received a home bleaching treatment with 10% carbamide peroxide. Before beginning the bleaching treatment, the selected patients responded a form with the Oral Health Impact Profile short form (OHIP-14) and Oral Aesthetic Subjective Impact Scale (OASIS) questionnaire. One month after the bleaching treatment, the patients answered the same questionnaires again. The mean ΔE after bleaching was obtained for the canines and lateral and central incisors. The OHIP-14 and OASIS data were measured for the total sample before and after bleaching using the Wilcoxon signed-rank test with a 5% significance level.

**Results:**

The effectiveness of whitening by significant color variation was observed in all groups of teeth with less variation in ΔE for central incisors. Comparing before and after bleaching, there was no significant difference in impact for any of the seven domains and total OHIP-14 scores, although a decrease was observed in the discomfort and psychological disability domains. As for the self-perception of dental aesthetics, measured by the OASIS, there was also no significant difference for all the items evaluated; however, it was possible to observe an increase in “concern with dental appearance” after bleaching.

**Conclusions:**

Home bleaching with 10% carbamide peroxide did not have a significant impact on patients’ quality of life and aesthetic perception, although there was a decrease in the domain of psychological discomfort and an increase in concerns about dental appearance.

** Key words:**Tooth Bleaching, quality of life, self-perception, dental aesthetics.

## Introduction

Currently, the population has become increasingly concerned about their aesthetic body and facial appearance, including that of aligned teeth, without blemishes and white color (1). Studies carried out in the United States and United Kingdom reveal that between 20 and 35% of the population recognize the staining of their teeth and are dissatisfied with the color they present (2,3), which has led to an increasingly frequent search for treatments for teeth bleaching, especially among the younger population (4) . The advantage of teeth bleaching lies in those treatments in which the dental structures are preserved. In addition, with the advent of new bleaching agents, the procedure is relatively quick and safe (5,6).

Traditionally, this procedure consists of applications of hydrogen peroxide or carbamide, in concentrations ranging from 6% to 38% and performed in the office or home and under the guidance of a professional (6). Due to its low molecular weight, the bleaching agent can penetrate the enamel and dentin. The whitener acts mainly through the oxidation of the organic compounds present in the dental structure, which are converted into carbon dioxide and water. When in contact with hard tissues, they release oxygen and free radicals that oxidize the pigments. The released oxygen penetrates the dentinal tubules and acts by breaking the long chains of the carbon rings that are highly pigmented into smaller chains and, consequently, giving the impression of lighter structures (7).

Oral health is related to the physical, psychological, and social well-being of individuals, which demonstrates the importance of the positive effects of dental treatments on people’s quality of life (8). Unsatisfactory oral conditions can affect social relationships, emotional well-being, as related to anxiety, insecurity, low self-esteem, and impaired dental function and speech. Thus, it is relevant to measure the impact of oral treatments on the daily lives of individuals (9), including teeth bleaching on patients’ general and local well-being.

Aesthetics is a subjective and variable perception among individuals and the assessment of its impact on quality of life is essential. Some instruments were developed in order to assess how much oral health is able to impact the quality of life of individuals (10-14), including the Oral Health Impact Profile (OHIP), which measures the discomfort and disability attributed to oral conditions (13), and the Oral Aesthetic Subjective Impact Scale (OASIS), which assesses an individual’s self-perception in relation to their oral aesthetics (15).

Since dental aesthetics are currently considered a primary factor in people’s social professional, and emotional relationships, it is pertinent that the dentist is aware of the aspects related to tooth bleaching therapy that can positively and negatively impact the quality of life and the perception of their patients. The exploration of these aspects is essential to guide the professional’s clinical conduct with the patient; thus assist in the choice of the bleach agent for bleaching efficiency and minimal sensitivity, and how to reinforce the role of dental hygiene for the longevity of the effects of the treatment.

The aim of this study was to evaluate the impact of home-made tooth bleaching with 10% carbamide peroxide on the patients’ quality of life and aesthetic perception. The null hypothesis to be tested: that there is no significant difference in the quality of life and aesthetic perception of patients before and after the bleaching treatment.

## Material and Methods

-Ethical aspects

This study was approved by the Research Ethics Committee (# 1,422,841). The study sample consisted of 107 patients who signed the informed consent form.

-Inclusion and exclusion criteria

The study included 107 individuals between 18 and 38 years of age, nonsmokers, who presented good oral and general health; all upper and lower anterior teeth (premolars, canines, and incisors) with pulpal vitality, free of caries lesions and restorations, without non-carious cervical lesions, without periodontal disease. Patients should have had at least one of the anterior teeth in color A2 or darker, based on the Vita Classic scale (Vita Zahnfabrik, Bad Säckingen, Germany).

The patients who were excluded were those who had: underwent any type of previous bleach procedure; stained teeth caused by tetracycline, fluorosis, gingival hyperplasia, or endodontic treatment; gingival retraction; dental sensitivity; parafunctional habits such as bruxism; used previous dental prostheses; used medications that contained heavy metals, analgesics, and anti-inflammatories prior to the bleaching treatment; or been pregnant or lactating.

-Experimental Design

This was a clinical study in which all patients received a home teeth bleaching treatment with 10% carbamide peroxide (Whiteness Perfect; FGM, Joinville, SC, Brazil) for 4 hours daily during 3 weeks. Before beginning the whitening treatment, all selected patients responded a form with the OHIP-14 and OASIS questionnaire. One month after the bleaching treatment, the patients answered the OHIP-14 and OASIS questionnaires again.

-OHIP-14 and the OASIS questionnaire

The OHIP-14 was used to measure the impact of bleaching on patients’ quality of life. This instrument was translated and validated for Brazilian Portuguese (16) and contains 7 domains (functional limitation, physical pain, psychological discomfort, physical disability, psychological disability, social disability, and disability), each with 2 items. The response categories are: never [0], rarely [1], sometimes [2], often [3], and always [4]. The total OHIP-14 score was obtained through the additive method by summing together the response codes of all items. The total score ranges from 0 to 56, with the highest score indicating a worse quality of life related to oral health. The frequency of impact was measured before bleaching and 30 days after bleaching.

The self-perception of oral esthetics was obtained through the OASIS, translated and validated in Brazil by Pimenta and Traebert (15). It consists of 5 questions related to the concern with self-perception of oral appearance, answered on a 7-point Likert-type scale, whereas 1 equals “never” and 7 equals “always” In this study, the instrument was answered by the participants before and 30 days after bleaching.

-Study intervention

The bleaching agent used was 10% carbamide peroxide. Before bleach treatment, all patients received prophylaxis with pumice and water; supragingival periodontal scraping sessions were performed for those with dental calculus. Individual molded alginate acetate trays for bleaching were made. The patients had their teeth color checked with the Vita Classic color scale (Vita Zahnfabrik, Bad Säckingen, Germany). After the trays were made, the patients were instructed to place a small drop of the product in each space in the tray for the teeth from the second premolar to the second premolar, upper and lower, and to use the bleaching gel every day, 4 hours per day, for 21 days. To evaluate the lightening, the period of saturation and color stabilization was waited. The effectiveness of whitening was assessed objectively using the VITA Easyshade Advance 4.0 spectrophotometer (Vivadent, Brea, CA, USA) by the same evaluator, before bleaching and 30 days after bleaching. The measurements were performed on the central and lateral incisors and upper canines and its value was calculated based on the x, y, and z chromaticity coordinates recommended by the International Commission Eclairage (CIE - 1976) (17), obtaining the CIE Lab color space in which L * represents brightness, a * and b * hue values. The difference in color perception between the 2 samples (ΔE) indicated the amount of color change that exists; the greater the value, the greater the lightening. To measure these color differences, the formula recommended by the CEI Lab method was used: ΔE = √ (L1-L2)2+ (a1-a2)2+ (b1-b2)2.

-Statistical analysis

The mean ΔE after bleaching was obtained for the canines and lateral and central incisors. The OHIP-14 and OASIS data were measured for the total sample before and after bleaching (Wilcoxon signed-rank test). The level of significance adopted for the analyses was 5%. The analyses were conducted using the Statistical Package for Social Sciences (IBM SPSS, version 21.0, IBM Corporation, Armonk, New York, USA).

## Results

The sample consisted of 107 patients, 36.4% males and 63.6% females, aged between 17 and 38 years, with the majority (62.6%) aged between 17 and 23 years (Table 1).

**Table 1:**
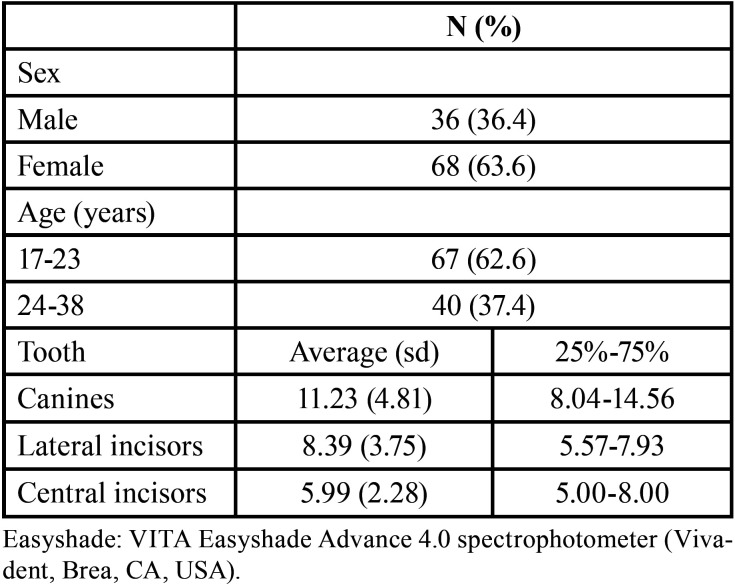
Sex, age, and average ΔE (Easyshade) after tooth whitening for canines, lateral and central incisors.

For ΔE, the lowest mean was observed for central incisors (Table 1). Comparing before and after bleaching, there was no significant difference in impact for any of the 7 domains and total OHIP-14 scores as well as for the instrument items (Table 2, Table 3).

**Table 2:**
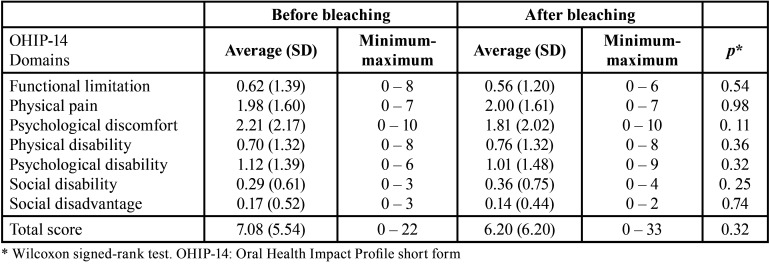
OHIP-14 total and domains scores for the total sample (107 adults), before and after home dental bleaching.

**Table 3:**
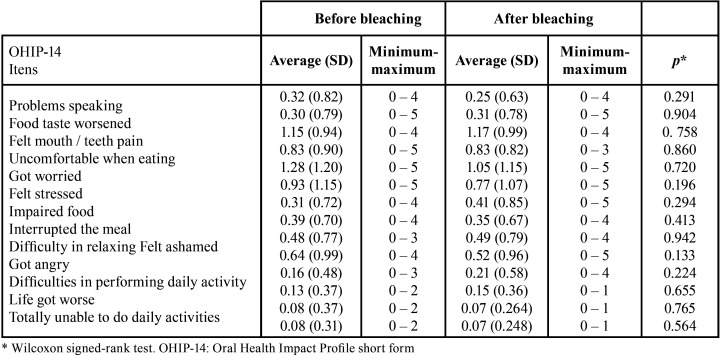
Score of OHIP-14 items for the total sample (107 adults), before and after home dental bleaching.

Regarding self-perception of dental aesthetics, as measured by the OASIS, no significant difference was found between before and after bleaching for all items. The highest mean score was presented for the item “concern about dental appearance” (Table 4).

**Table 4:**
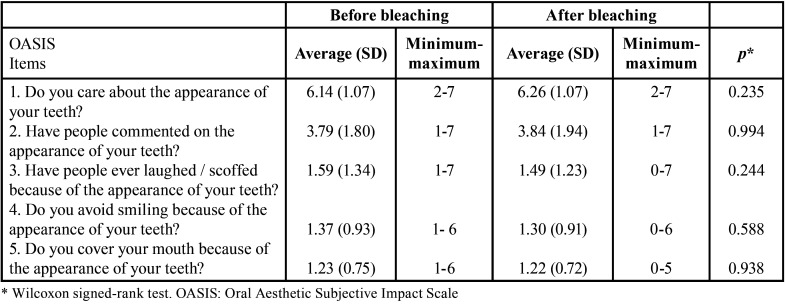
OASIS items scores for the total sample (107 adults) before and after home dental bleaching.

## Discussion

The effectiveness of tooth bleaching was observed objectively by the color variation obtained for ΔE; it was observed that all the teeth evaluated obtained a positive color variation compared after the whitening treatment. There was a decrease in the average impact on individuals’ daily lives, although without statistical significance. Our findings corroborate a systematic review that found a lack of association between tooth whitening and positive impacts on quality of life related to oral health (18).

In this study, the most representative mean scores were for the domains related to emotional (Psychological discomfort and disability) and, specifically, for items related to concern about teeth or their appearance. Many studies have shown that patients consider tooth color to be a determining factor in their satisfaction with dental appearance (19,20). This indicates that dental discoloration can decrease a patient’s self-realization, resulting in harmful effects to their emotional state (21), demonstrating that in addition to physical health, mental health has profound implications for the quality of life of individuals. Physical pain associated with diseases and dental disorders can impact an individual’s daily living activities as well as cause feelings of shame about unattractive teeth, which tends to decrease social interactions in older adults (22).

Bleaching treatment can generate positive and/or negative impacts on patients’ quality of life (23). The positive effects are generally related to changes in the color of the teeth, which reflect in a favorable dental aesthetic self-perception. The negative effects are related to the sensitivity and discomfort caused by the bleaching agents (24), usually when higher concentrations of the bleaching agent are used.

In the present study, no significant negative effects, such as pain and function limitations, were found, probably due to the low concentration of the bleaching agent and the absence of gingival irritation. In relation to the positive effects, the lack of significant findings presumably occurred due to discrete color changes conferred by the treatment or by the gradual whitening process that the home procedure provided, thus unnoticed by patients and leading them to having underestimated the degree of the lightening effect.

Increasingly, subjective aspects such as patients’ feelings and opinions have been added to the objective instruments for evaluating treatments, with the patient’s perception of satisfaction being a highly evaluated parameter (25). For the tooth whitening procedure, it is not different because the well-being of the patients who seek an aesthetic treatment is their perception of success.

The absence of significant negative or positive impacts in the present study corroborates with the findings of an other study in which in-office dental bleaching was bleahed with low concentration hydrogen peroxides (26). In this study, the treatments proved to be effective, with low levels of tooth sensitivity and gingival irritation, and without affecting the participants’ quality of life. Thus, it is possible to affirm that the concentration of the product is a determining factor for the negative effects. It is worth mentioning that in the face of negative effects such as sensitivity, its occurrence is usually transient and of short duration, but it can cause a negative psychological effect when the patient associates the bleaching procedure with the possibility of pain, and can cause the withdrawal from or interruption of treatment. (27). Thus, it is important that the professional is aware of the potential possibility of the impact of tooth sensitivity and guides his patients regarding behaviors that can avoid or mitigate the negative effects that may arise as a result of the whitening treatment.

Assessing oral health-related quality of life is complex and covers different domains. In addition to subjectivity, age can directly influence aesthetic and sensory aspects such as pain. A study demonstrated that the elderly persons are more resilient and reported less negative impacts when aesthetics and tooth sensitivity due to hot, cold, and sweet stimuli were evaluated (28). However, younger individuals are more concerned with tooth alignment and color (29) and, therefore, can influence the observation of a “positive” impact of whitening treatment. These positive changes in dental aesthetics allow patients to improve their interactions with the environment and with people, since aesthetic dental problems tend to profoundly affect the social and emotional aspects of people’s lives (30).

The planning of the bleaching treatment to obtain the desired aesthetic effect and minimum care in order to avoid the negative impacts of sensitivity should be observed by the professionals. In addition, it is important that future studies address the impact and perception of patients comparing other types of whitening treatments, since quality of life related to oral health is an important part of the overall assessment of treatment.

Home bleaching with 10% carbamide peroxide did not have a significant impact on patients’ quality of life and aesthetic perception, although from the data of the OHIP-14 there was a decrease in the domain of psychological discomfort, and from the data of the OASIS there was an increase in concerns about dental appearance in the study population.
